# Health system adaptations for extreme heat: Protocol for an international scoping review of reviews

**DOI:** 10.1371/journal.pone.0307417

**Published:** 2024-07-18

**Authors:** John Richmond, Mark Clowes

**Affiliations:** School of Medicine and Population Health, University of Sheffield, Sheffield, South Yorkshire, United Kingdom; Clinton Health Access Initiative, UNITED STATES OF AMERICA

## Abstract

**Objective:**

The objective of this study is to map the international evidence for extreme heat related adaptation strategies by health systems, with a particular focus on how heat-vulnerable populations and local situational awareness are considered in these strategies.

**Introduction:**

Since the Paris Climate Accords in 2015, awareness has increased of the health risks posed by extreme heat along with interest in adaptations which aim to reduce heat-health-risks for vulnerable populations. However, the extant literature on these adaptations suggest they are insufficient, and call for research to examine whether, how, and what adaptations for extreme heat are effective as public health interventions.

**Inclusion criteria:**

We will include English-language review articles describing and/or evaluating health system adaptations for extreme heat. Health systems will be defined broadly using the WHO Building Blocks model [1] and adaptations will range from the individual level to institutional, regional and national levels, with particular attention to localisation and the protection of vulnerable individuals.

**Methods:**

A comprehensive literature search of the published literature will be conducted using MEDLINE, Embase, CINAHL, the Cochrane Library and Web of Science. Searches will be limited to reviews published since 2015 in the English language. Results will be exported to EndNote for screening (with a sample checked by two reviewers to ensure consistency). A complementary search for related reports by major international agencies (e.g. WHO; International Association of Emergency Managers), as well as local searches for current guidance and case studies, will be conducted in parallel. Data from included papers will be presented in tables with a narrative commentary.

## Introduction

In this scoping review of reviews, we aim to map the evidence for extreme heat related adaptation strategies by health systems with particular focus on how heat-vulnerable populations are considered.

Global warming and climate change is positively correlated with human activity, not limited to burning fossil fuels and unsustainable energy use, leading experts to predict global surface temperatures will increase by 1.5°C in the first half of the 2030s [2]. Climate change is driving an increase in the frequency, intensity, and duration of extreme weather events, such as wildfires, floods, and heatwaves [3]. These events are known to have an impact on health systems, including driving hospital admissions and length of stay, over heating facilities, and worsening symptoms for people with existing health conditions [4]. The COVID-19 pandemic has shown us that many health systems were ill prepared to cope with the additional pressures that result from such a prolonged emergency. As a result, it has become increasingly common for health systems to intervene by enhancing disaster preparedness and planning for extreme weather emergencies [3]. This review protocol focuses specifically upon health system adaptation strategies for extreme heat events.

Extreme Heat events are defined as a period of sequential warm days, often referred to as a heat wave, where the maximum and minimum temperatures are unusually high and exceed a threshold set by local governments which vary by region, for example in the United Kingdom these thresholds range between 25°C and 28°C [5–7]. These events impact infectious disease transmissions and undermine people’s mental health and livelihoods [8]. This increase in temperature combined with land use changes are making more regions suitable for the transmission of vector-borne diseases, for example increasing rates of dengue in Central and South America [8]. Exacerbation of existing mental health symptoms during extreme heat can lead to increased risk of suicide among vulnerable individuals. Livelihoods are affected as certain commercial activities can put workers at risk, especially those who work outside during extreme heat [9].

Globally, deaths during a heatwave are most likely to occur among vulnerable populations, we define this as: the very young (<5), and older people (>65) who live in their own homes, care homes, or are admitted to the hospital [10]. Heat-vulnerable communities include those with existing mental health concerns, metabolic syndrome, and from ethnic minority communities [11, 12]. Increased demand upon health services is a problem in extreme heat, particularly among underserved groups. Vulnerability to heat events is compounded by inequity, intersectionality and marginalisation with variation by geographic region. Several types of adaptation strategies have been implemented by health systems to mitigate these health risks.

Adaptation strategies are defined by the Interplanetary Commission on Climate Change (IPCC) as “the process of adjustment to actual or expected climate and its effects in order to moderate harm or take advantage of beneficial opportunities” [8]. It has been suggested that adaptations could reduce pressures on healthcare services and deliver £10 in economic benefits for every £1 invested [13]. However, recent reviews [14] flag that current adaptations are insufficient, do not adequately consider health equity, and require better coordination between organisations. The consequences of this current adaptation approach are that many interventions, such as early-warning systems and behavioural risk messaging, do not reach their intended targets (e.g. vulnerable groups), due to barriers including cultural differences, language, and a lack of trust, particularly among ethnic minority populations.

Re-defining who is considered heat-vulnerable is important for targeted adaptations to be effective. Interconnected factors influence vulnerability to extreme heat including age, pre-existing medical conditions, disability, socioeconomics, and the built environment. Addressing heat exposure by individuals in workplaces (e.g. working outside) is another challenge. This scoping review aims to expand understanding of who is considered heat-vulnerable; why; and how to reach them effectively, by examining targeted policies and actions to mitigate the adverse effects of extreme temperatures on at-risk populations.

Moreover, we aim to consider how local situational awareness is incorporated with adaptation so that risk communication plans are grounded in local knowledge and experience. We define local situational awareness as the development of a shared understanding of health needs in local communities, cross-jurisdictional information sharing and enhanced coordination, to ensure the implementation of adaptation strategies are targeted and effective [15]. For example, research finds that messages about the dangers of hot weather are not getting through to certain heat-vulnerable communities [11] We will highlight examples of how tailored messaging (e.g. using diverse channels; languages; and formats; or incorporating individuals from heat-vulnerable communities) could build trust with message receivers and increase the reach of heat risk education, reducing inequities in health outcomes.

### Review questions

1. What adaptations are health systems worldwide undertaking in the face of extreme heat emergencies caused by climate change?
2. How are heat-vulnerable populations considered in these adaptations?
3. How is local situational awareness incorporated with adaptation, so that strategies are grounded in local knowledge and experience?

## Eligibility criteria

### Population / participants

We define international health systems using the Health Systems Framework laid out by the WHO [1], including service delivery, health workforce, information, medical products & technologies, financing, leadership/ governance. As such, we consider health system as consisting of all the organizations, institutions, resources and people whose primary purpose is to improve health. This includes initiatives which aim to influence determinants of health, as well as more direct health-improvement activities. Public health actions which deliver preventative, promotive interventions as well as healthcare facilities which deliver curative and rehabilitative interventions by both state and non-state actors are included.

### Concept

Adaptations may be implemented by individuals; by organisations; or by governments or public health authorities (at local, regional or national level). Kiarsi et al. [16] identified 11 categories of adaptive behaviour and this is the conceptual framework we will use to classify interventions described in our included evidence.

### Context

While extreme heat events can occur due to changes in global weather patterns, the increase in the frequency, duration, and intensity of these events over the last several decades is linked to the warming of the planet and attributed to human activity [17]. While some parts of the world are more accustomed to dealing with this kind of hot weather than others, it is challenging those that lack infrastructure to cope with it; and certain locations are seeing extended seasonal peaks of significantly above normal thresholds.

For the purpose of this review, we will include reviews which describe how health systems are adapting to extreme heat, and to the secondary hazards which are directly and indirectly related, such as wildfires and droughts [18].

### Types of evidence sources

Reviews published in English language since 2015 (the date of the Paris Accord) will be included.

An additional, parallel search will be conducted for recent reports by major international agencies (e.g. WHO; International Association of Emergency Managers, United Nations) plus current guidance and case studies from international health systems represented by members from our Health System Resilience for Extreme Weather Emergencies Network [19].

## Methods

The proposed scoping review will be conducted in accordance with the PRISMA-ScR standards for reporting scoping reviews [20].

### Identification of studies

The search strategy, designed and executed by an information specialist (MC) will locate published review articles through a comprehensive search of the following databases:

- MEDLINE (including Medline-in-process and Epub ahead of print)
- Embase
- CINAHL
- The Cochrane Library
- Web of Science

A full search strategy for MEDLINE has already been developed (see S1 Appendix). This search strategy, including all identified keywords and index terms, will be adapted for each included database and/or information source. Informal grey literature searches will conducted by members of the network to identify evidence from their own local health systems.

### Screening

Following the search, all identified citations will be collated and uploaded into EndNote and duplicates removed. Following a pilot test, titles and abstracts will be screened for assessment against the inclusion criteria for the review (with a sample double-screened to check agreement). Potentially relevant sources will be retrieved in full text for assessment in detail, and reasons for exclusion of sources of evidence at this stage be recorded and reported in the scoping review. Any disagreements that arise between the reviewers at each stage of the selection process will be resolved through discussion, or with an additional reviewer/s. The results of the search and the study inclusion process will be reported in full in the final scoping review and presented in a Preferred Reporting Items for Systematic Reviews and Meta-analyses extension for scoping review (PRISMA-ScR) flow diagram [20].

### Eligibility criteria (inclusion / exclusion)

Review articles will be eligible for inclusion if they:

- have used systematic (or partially systematic) processes
- address heat-related events caused by climate change
- discuss adaptations to increase resilience/preparedness in any part of the health system
- are published in the English language

If review-level evidence is scarce for particular countries or types of intervention, we may additionally include primary studies and/or grey literature.

### Data extraction

Data will be extracted from papers included in the scoping review using a data extraction tool (a custom-designed Excel spreadsheet) developed by the reviewers. The data extracted will include specific details about the participants, concept, context, study methods including a categorisation of the types of adaptation, hazard(s) and/or populations addressed, and at which level of the health system they are implemented. The draft data extraction tool will be modified and revised as necessary during the process of extracting data from each included evidence source.

Included reviews will not be subject to critical appraisal although we will record whether they followed systematic or quasi-systematic methods.

### Data analysis and presentation

Data from the included reviews will be presented in tabular form along with a commentary and narrative summary of the findings, including features of the literature such as types of intervention discussed; geographical coverage; and areas where evidence is scarce.

## Ethics and dissemination

This study does not require ethical approval as it is a scoping review protocol. The findings will be published in scientific journals and disseminated electronically. All relevant literature search results such as government documents, journal articles, and publications, will be made available in a public repository.

## Strengths and limitations of this study

The breadth of this research question (the range of potential adaptation interventions at different levels of the health system; plus the global coverage) necessitated the focus on review-level literature, to the exclusion of primary studies. We recognise this as a limitation; however, our survey of current grey literature from our network partners is designed to mitigate the associated risks by bringing the review up to date. We believe the unique contribution of our review will be to provide a helicopter view of the international literature to identify where there are gaps for further research or more focused systematic reviews.

## Supporting information

S1 Appendix Search strategy. (DOCX)

S2 Appendix PRISMA-P checklist. (DOCX)
