# Supplementary material for: Health system adaptations for extreme heat: Protocol for an international scoping review of reviews
Source: PLoS One. 2024 Jul 18;19(7):e0307417. doi: 10.1371/journal.pone.0307417 (PMC11257315; doi:10.1371/journal.pone.0307417)
Supplement: S1 Appendix — (DOCX) [file pone.0307417.s001.docx]

## S1 APPENDIX – search strategy

Ovid MEDLINE(R) ALL <1946 to August 07, 2023>

1 ((climat* or environment*) adj2 (chang* or disaster* or catastroph* or emergenc*)).mp. 109093

2 exp Climate Change/ 30214

3 (global warm* or (ris* adj3 temperature*)).mp. 26690

4 (el nino or la nina).mp. 3805

5 1 or 2 or 3 or 4 131094

6 (wild fire* or wildfire* or forest fire* or bush fire*).mp. 4936

7 (heatwave* or heat wave* or extreme heat* or drought* or heat island* or urban heat*).mp. 39052

8 ((heat adj2 (stroke or exhaust*)) or hypertherm* or hyper-therm* or overheat* or over-heat* or dehydrat* or de-hydrat*).mp. 113080

9 6 or 7 or 8 153325

10 EPRR.mp. 15

11 ((heat* or hot or weather or temperature* or climate) adj2 (resilien* or adapt* or mitigat* or plan or plans or planning or initiative* or intervention*)).mp. 11756

12 ((disaster* or emergenc* or cris#s or incident*) adj2 (manag* or prepar* or simulat* or resilien* or plan* or train* or contingenc* or ready or readiness or readyness or hardyness or hardiness or prevent* or respon*)).mp. 51269

13 ((public adj2 inform*) or (health adj2 (promot* or educat*)) or (aware* adj2 (public or rais*))).mp. 268167

14 (ventilat* or cool* or fan or fans or spray* or self-care or self-monitor* or water supplementation or cotton or shade or shading or reflective paint or heat proof* or climate proof* or cooling shelter* or cooling cent* or tree canop* or "green and blue" or water feature* or pond* or mechanical cooling or air duct*).mp. 488533

15 incident commander*.mp. 58

16 emergency operations cent*.mp. 160

17 exp Risk Assessment/ or exp Disaster Planning/ or exp Risk Reduction Behavior/ or exp Risk Management/ 374357

18 (((impact* or demand) adj2 manag*) or surge capacity).mp. 7090

19 ("early warning" or "warning system*" or surveill* or monitor*).mp. 1448176

20 10 or 11 or 12 or 13 or 14 or 15 or 16 or 17 or 18 or 19 2482560

21 (((health* or medical) adj2 (facilit* or centre* or center* or system* or service* or organi#ation* or workforce* or work force* or information system* or leadership or governance)) or hospital* or emergency room or emergency ward* or primary care or general practi*).af. 9383740

22 (public health or policy or policies or government* or national or federal or district* or region*).ti,ab. 3135985

23 21 or 22 11383087

24 5 and 9 and 20 and 23 1729

25 limit 24 to (yr="2015 -Current" and "reviews (best balance of sensitivity and specificity)") 185

26 (5 or 6 or 7) and 20 and 23 15772

27 limit 26 to (yr="2015 -Current" and "reviews (best balance of sensitivity and specificity)") 1501

28 5 and 9 and 20 3406

29 limit 28 to (yr="2015 -Current" and "reviews (best balance of sensitivity and specificity)") 357

30 25 or 27 or 29 1673

31 5 and 9 and (21 or "public health".mp.) 1361

32 limit 31 to (yr="2015 -Current" and "reviews (best balance of sensitivity and specificity)") 260

33 30 or 32 1835

34 remove duplicates from 33 1829
